# Prognostic value of preoperative inflammatory markers in patients with hepatocellular carcinoma who underwent curative resection

**DOI:** 10.1186/s12935-021-02204-3

**Published:** 2021-09-17

**Authors:** Wenlong Wu, Quancheng Wang, Dandan Han, Jianhui Li, Ye Nie, Dongnan Guo, Long Yang, Kaishan Tao, Xuan Zhang, Kefeng Dou

**Affiliations:** 1 Department of Hepatobiliary Surgery, Xijing Hospital, The Fourth Military Medical University, Xi’an, 710032 Shaanxi China; 2 School of Pharmacy, Health Science Center, Xi’an Jiaotong University, Xi’an, 710061 Shaanxi China

**Keywords:** Hepatocellular carcinoma, Platelet-to-lymphocyte ratio, Gamma-glutamyl transpeptidase-to-platelet ratio, Aminotransferase-to-lymphocyte ratio, Prognosis

## Abstract

**Background:**

The prognosis of hepatocellular carcinoma (HCC) is not optimistic. Our study focused on present inflammatory markers, including the neutrophil-to-lymphocyte ratio (NLR), platelet-to-lymphocyte ratio (PLR), gamma-glutamyl transpeptidase-to-platelet ratio (GPR), aspartate aminotransferase-to-lymphocyte ratio (ALR) and fibrinogen-to-albumin ratio (FAR), and explored their optimal combination for the prognosis of HCC after resection.

**Methods:**

A total of 347 HCC patients who underwent curative resection were enrolled. The optimal cutoff values of the inflammatory markers were calculated using receiver operating characteristic (ROC) curve analysis, and used to divide patients into two groups whose differences were compared by Kaplan–Meier analysis. Cox univariate and multivariate analyses were used to analyze the independent prognostic inflammatory markers. The χ^2^ test was chosen to determine the relationship between independent prognostic inflammatory markers and clinicopathological features. We created combined scoring models and evaluated them by Cox univariate and multivariate methods. The concordance index (C-index), Akaike information criterion (AIC) and likelihood ratio were calculated to compare the models. The selected optimal inflammatory markers and their combinations were tested in different stages of HCC by Kaplan–Meier analysis.

**Results:**

The ALR and GPR were independent prognostic factors for disease-free survival (DFS); the ALR, PLR, and GPR were independent prognostic factors for overall survival (OS). The proposed GPR and ALR-GPR-PLR score models were independent predictors for DFS and OS, respectively.

**Conclusion:**

The preoperative GPR and ALR-GPR-PLR score models were independent predictors for DFS and OS, respectively, and performed well in stratifying patients with HCC. The higher the score in the model was, the worse the prognosis.

**Supplementary Information:**

The online version contains supplementary material available at 10.1186/s12935-021-02204-3.

## Background

Primary carcinoma of the liver, known as one of the most commonly diagnosed cancers and the third leading cause of cancer death worldwide, includes intrahepatic cholangiocarcinoma and hepatocellular carcinoma (HCC). More than 50% of HCC cases are in China [1, 2], and HCC has attracted increasing attention. Liver resection is still the mainstay of treatments for HCC patients. However, the clinical prognosis of HCC remains poor despite advances in diagnostic and surgical techniques [3].

Within 5 years after curative resection, more than half of patients with HCC relapse or exhibit metastases [4]. The Barcelona Clinic Liver Cancer (BCLC) staging system, including recommendations for treatments, has been widely validated and is the most frequently used staging method [5, 6]. The American Joint Committee on Cancer (AJCC) and China Liver Cancer (CNLC) staging systems are also commonly considered [7, 8]. These staging systems have some limitations due to the heterogeneity of tumors. In addition, plasma alpha-fetoprotein (AFP) levels, a common tumor marker for HCC, remain within the normal range in 15–30% of advanced HCC patients [9]. Therefore, more efficient prognostic indicators need to be explored to conduct active interventions to improve survival rates.

Inflammation promotes tumorigenesis and the development of cancers [10]. In China, chronic hepatitis B virus (HBV) infection is the major pathogenic factor for HCC, whereas in Western countries, hepatitis C virus (HCV) infection accounts for the main risk factors [8]. In recent years, some inflammatory indexes have been suggested to predict the prognosis of HCC. For example, the neutrophil-to-lymphocyte ratio (NLR) and platelet-to-lymphocyte ratio (PLR) have been reported as novel prognostic biomarkers for gastric cancer, colon cancer, cervical cancer, ovarian cancer and HCC [11–17]. In addition, the gamma-glutamyl transpeptidase-to-platelet ratio (GPR) has been suggested to be related to HCC prognosis [18, 19]. Furthermore, other inflammatory markers, such as the aspartate aminotransferase-to-lymphocyte ratio (ALR) and fibrinogen-to-albumin ratio (FAR), have been suggested to predict the prognosis of HCC [20–22].

Although the above indexes have prognostic value, it is unclear which marker has a better predictive role in HCC. In addition, it is worth exploring whether the combined use of these indicators can improve the accuracy of the postoperative prognosis of HCC. Few studies have compared these inflammatory indicators in predicting prognosis after patients with hepatocellular carcinoma undergo curative resection. Therefore, it will be of great significance to establish moderate models for the prognosis of HCC using these inflammatory markers.

Our study focused on comparing the effects of the NLR, PLR, GPR, ALR and FAR on the prognosis of HCC patients who had undergone liver resection, to find the optimal combination and to establish models that can accurately predict prognosis.

## Methods

From January 1st, 2014 to December 31st, 2017, 394 HCC patients with complete clinical materials and follow-up data who underwent hepatectomy were included. The inclusion criteria were as follows: (1) pathological diagnosis of HCC; (2) surgical resection performed as the first treatment; (3) curative resection with a negative surgical margin; (4) at least 18 years old; and (5) complete preoperative laboratory examination data.

**Figure. 1:**
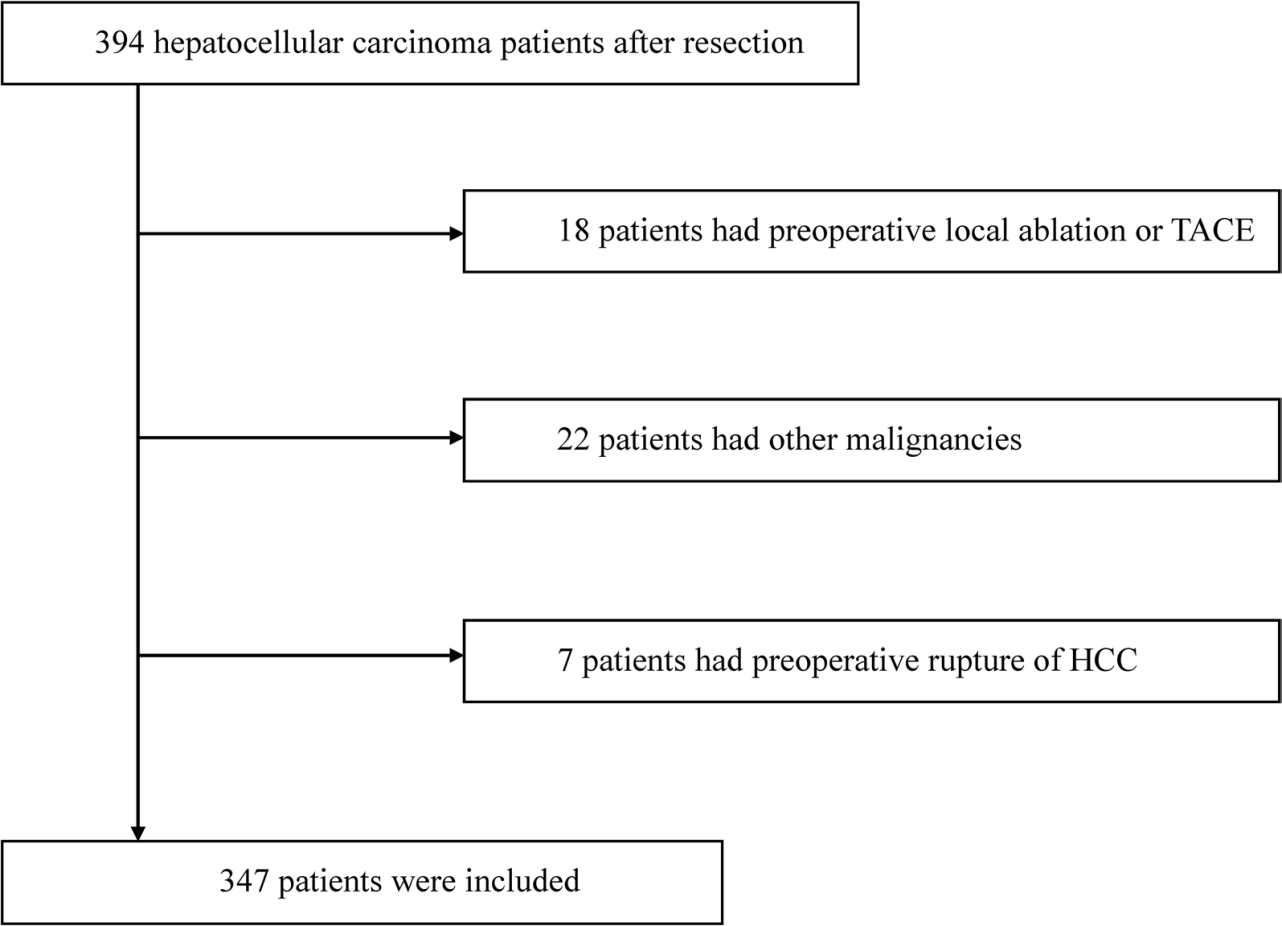
The flowchart of patient enrollment

The following exclusion criteria were used: (1) surgical treatment, local ablation, transarterial chemoembolization (TACE) or radiotherapy before hepatectomy; (2) other malignant tumors, AIDS, recent acute infection, or high fever; (3) recent administration of anti-inflammatory drugs or immunosuppressants; (4) rupture of HCC; and (5) incomplete clinical or pathological data. Finally, 347 HCC patients who underwent resection were included in this study. The flowchart of patient enrollment is shown in Fig. 1. Routine examinations were performed within one week before surgery: routine blood examination, blood biochemical series examination, chest X-ray, abdominal ultrasound, and computed tomography (CT) or magnetic resonance imaging (MRI). Clinical variables, including demographic data, medical history, complete blood count, albumin (Alb), AFP, aspartate aminotransferase (AST), gamma-glutamyl transpeptidase (γ-GT), total bilirubin (TB), prothrombin time (PT), fibrinogen, tumor pathological parameters, postoperative treatment, Child–Turcotte–Pugh class (Child class), Eastern Cooperative Oncology Group (ECOG) performance status (PS), BCLC staging, AJCC staging, and CNLC staging, were collected. The equations of the inflammatory markers NLR, PLR, GPR, ALR, and FAR are shown as follows:

NLR = neutrophil count/lymphocyte count ratio; PLR = platelet count/lymphocyte count ratio; GPR = γ-GT/platelet count ratio; ALR = aspartate aminotransferase/lymphocyte count ratio; FAR = fibrinogen/albumin ratio.

All the patients were followed up through the electronic medical record system of the hospital and phone calls every three months starting from the date of surgery. Follow-up contents included recent health status, medications taken, hematologic tests, AFP levels, abdominal ultrasound, CT and MRI results. If abnormalities were found, we recommended enhanced CT or MRI, accompanied by further tests to confirm the patients’ conditions. Recurrence was evaluated mainly according to imaging. Disease-free survival (DFS) was defined by the time from the date of liver resection to the date of relapse or the date lost to follow-up. Overall survival (OS) was defined by the time from the date of liver resection to the date of HCC-associated death or the date lost to follow-up. The patients received our best medical care.

## Statistical analysis

SPSS for Windows version 22 (SPSS, Chicago, IL, USA) was used to analyze most of the data in this study. Receiver operating characteristic (ROC) curves and the optimal cutoff values of the inflammatory markers were determined using MedCalc Statistical Software version 18.2.1 (MedCalc Software bvba, Ostend, Belgium). Optimal cutoff values were determined according to Youden's index. Continuous variables are presented as the median (P_25_ (lower quartile) – P_75_ (upper quartile)).

The optimal cutoff values of inflammatory markers were used to divide patients into two groups. The Kaplan–Meier method with log-rank test was chosen to measure the differences between two groups. The figures were drawn by GraphPad (GraphPad Software Inc. Prism Version 8.0.2, USA). Cox univariate analysis was performed for inflammatory markers and other common clinical prognostic indicators. The variables of univariate analysis with P < 0.1 were imported into the multivariate analysis. Cox multivariate analysis was performed with the “Forward LR” method, and P < 0.05 (two-sides) indicated statistical significance. The χ^2^ test was chosen to determine the relationship between independent prognostic inflammatory markers and clinicopathological features. Then, we created scoring models by combining the independent prognostic inflammatory markers. The combined scoring models integrating other common clinical prognostic indicators were analyzed by Cox univariate and multivariate analyses. To compare single inflammatory markers and the combined scoring models, R software (R Core Team (2020), Vienna, Austria) with the “survival” package (R package version 3.2-7, Terry M. Therneau, Patricia M. Grambsch (2000)) was used to calculate the concordance index (C-index) and likelihood ratio. The Akaike information criterion (AIC) was computed using the following formula: AIC = 2k − 2In(L).

The prediction accuracy, goodness of fit and uniformity of the inflammatory markers and the combined scoring models were compared by the C-index, AIC and likelihood ratio. The selected optimal inflammatory markers and combined scoring models were tested in different stages of HCC by Kaplan–Meier analysis.

## Results

### Demographics and tumor characteristics

The present study included 347 patients, of whom 83.6% (290 patients) were male and 16.4% were female (57 patients). Among the 347 patients, 300 patients’ (86.5%) hepatitis B surface antigen (HBsAg) test was positive, 222 patients (64%) had liver cirrhosis, 188 patients (54.2%) received postoperative local ablation therapy or TACE, and 33 patients (9.5%) used multiple kinase inhibitor (MKI). In terms of tumor parameters, 304 patients (87.6%) had a tumor capsule; 305 patients (87.9%) had a single tumor; 168 patients’ (48.4%) tumor size was > 5 cm; 172 patients (49.6%) had microvascular invasion (MVI); and 29 (8.4%), 283 (81.6%), and 35 (10.1%) patients had well, moderately and poorly differentiated tumor cells, respectively. To facilitate the analysis, 9 patients with BCLC stage 0 were merged into the BCLC stage A group, and 5 patients with AJCC stage IV were merged into the AJCC stage III group (Table 1).

**Table 1:** Baseline characteristics of the 347 patients

Variable		Patients (%)/median (P_25_–P_75_)^a^	Variable		Patients (%)/median (P_25_–P_75_)
Sex	Male	290 (83.6)	Child class	A	335 (96.5)
	Female	57 (16.4)		B	12 (3.5)
Age (years)	≥ 60	95 (27.4)	Performance status	1	144 (41.3)
	< 60	252 (72.6)		0	203 (58.2)
HBsAg	Positive	300 (86.5)	BCLC staging	A^d^	175 (50.4)
	Negative	47 (13.5)		B	17 (4.9)
Liver cirrhosis	Yes	222 (64)		C	155 (44.7)
	No	125 (36)	AJCC staging	I	166 (47.8)
Portal vein invasion	Yes	21 (6.1)		II	136 (39.2)
	No	326 (93.9)		III^e^	45 (13)
Ascites	Yes	11 (3.2)	CNLC staging	I	291 (83.9)
	No	336 (96.8)		II	33 (9.5)
Ablation or TACE	Yes	188 (54.2)		III	23 (6.6)
	No	159 (45.8)	Neutrophil count		3.6 (2.56–4.72)
AFP (ng/ml)	> 400	132 (38)	Lymphocyte count		1.45 (1.03–1.84)
	≤ 400	215 (62)	Platelet count		150 (109–205)
Tumor capsule	Yes	304 (87.6)	γ-GT		60 (36–115)
	No	43 (12.4)	AST		37 (27–53)
Tumor number	≥ 2	42 (12.1)	Total bilirubin		15.2 (12–21.2)
	1	305 (87.9)	Prothrombin time		11.7 (11–12.4)
Tumor size (cm)	> 5	168 (48.4)	Albumin		44 (40.7–46.7)
	≤ 5	179 (51.6)	Fibrinogen		2.61 (2.13–3.27)
MVI^b^	Yes	172 (49.6)	NLR		2.44 (1.71–3.37)
	No	175 (50.4)	PLR		104.46(74.69–150.52)
Cell differentiation	Poor	35 (10.1)	GPR		0.45 (0.22–0.88)
	Moderate	283 (81.6)	ALR		26.97 (17.02–44.74)
	Well	29 (8.4)	FAR		0.06 (0.05–0.08)
MKI^c^	Yes	33 (9.5)			
	No	314 (90.5)			

^a^P_25_–P_75:_ (lower quartile—upper quartile)

^b^MVI: microvascular invasion

^c^MKI: multiple kinase inhibitor

^d^Nine patients with BCLC stage 0 were merged into the BCLC stage A group

^e^Five patients with AJCC stage IV were merged into the AJCC stage III group

### Optimal cutoff values of the inflammatory markers

**Figure. 2:**
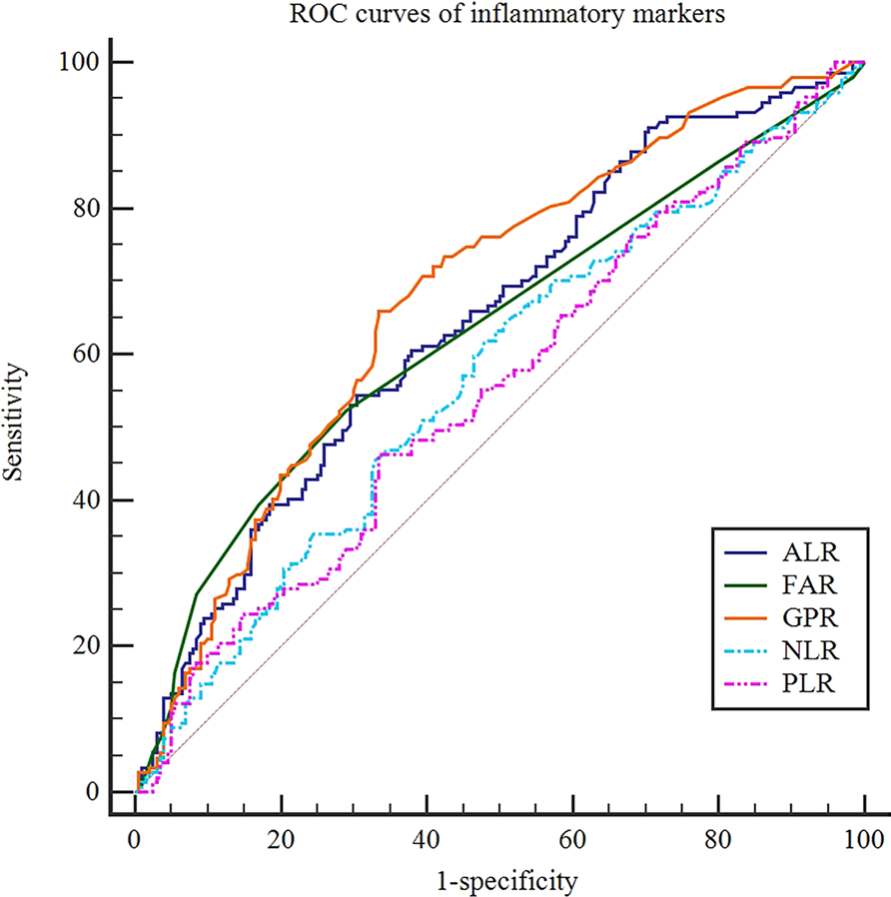
The ROC curves of NLR, PLR, GPR, ALR, and FAR

The optimal cutoff values of the NLR, PLR, GPR, ALR, and FAR were 2.33, 117.09, 0.48, 31, and 0.06, respectively. The areas under the curve (AUC) of those markers were 0.569, 0.553, 0.680, 0.647, and 0.632 (Fig. 2).

### OS and DFS rates

**Figure. 3:**
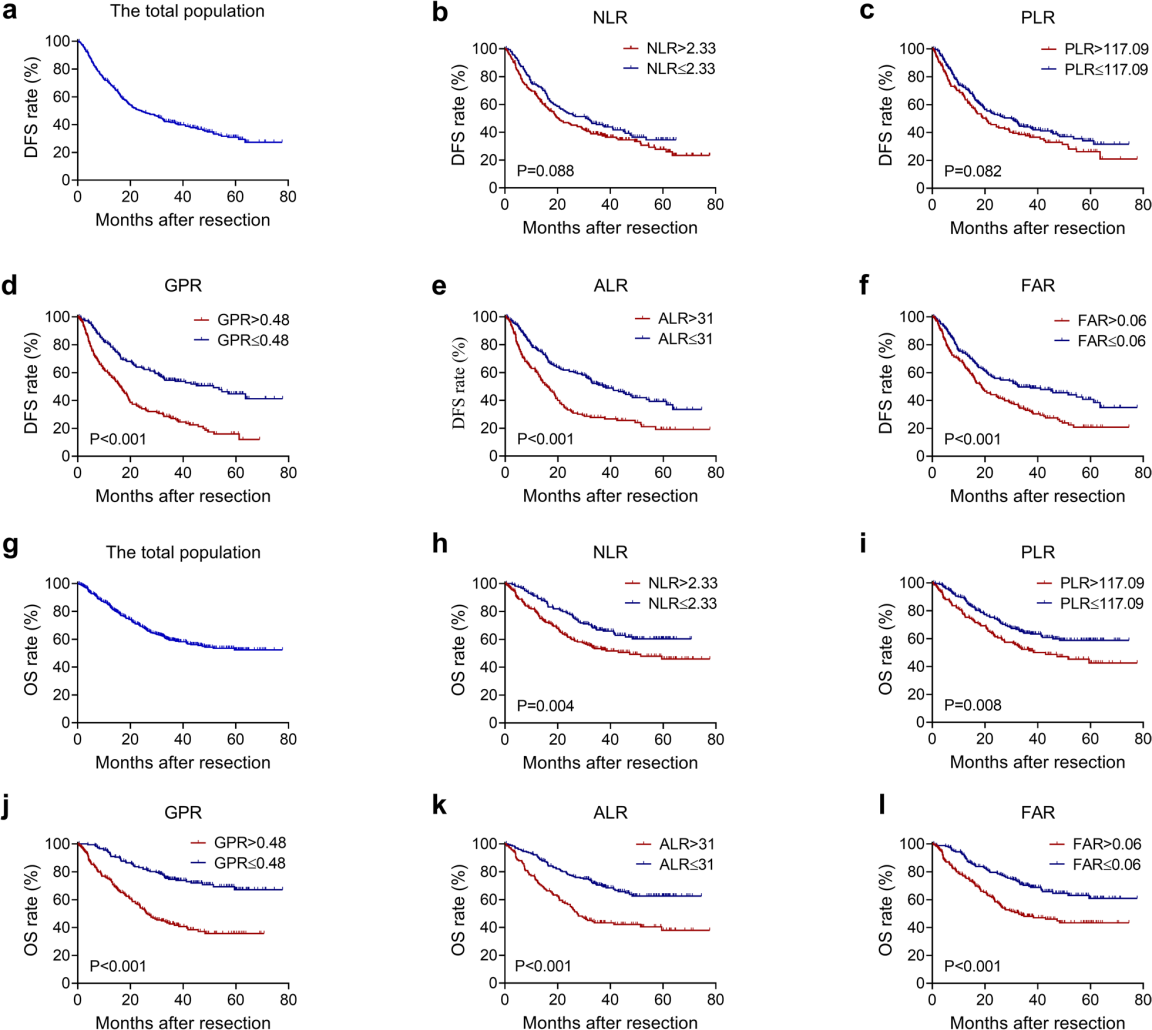
Kaplan–Meier analysis of (**a**) the total population(N = 347), (**b**) NLR(N_NLR>2.33_ = 188, N_NLR≤2.33_ = 159), (**c**) PLR(N_PLR>117.09_ = 135, N_PLR≤117.09_ = 212), (**d**) GPR(N_GPR>0.48_ = 165, N_GPR≤0.48_ = 182), (**e**) ALR(N_ALR>31_ = 141, N_ALR≤31_ = 206), and (**f**) FAR(N_FAR>0.06_ = 171, N_FAR≤0.06_ = 176) for DFS; Kaplan–Meier analysis of (**g**) the total population(N = 347), (**h**) NLR(N_NLR>2.33_ = 188, N_NLR≤2.33_ = 159), (**i**) PLR(N_PLR>117.09_ = 135, N_PLR≤117.09_ = 212), (**j**) GPR(N_GPR>0.48_ = 165, N_GPR≤0.48_ = 182), (**k**) ALR(N_ALR>31_ = 141, N_ALR≤31_ = 206) and (**l**) FAR(N_FAR>0.06_ = 171, N_FAR≤0.06_ = 176) for OS

The median follow-up time was 45 months. During the follow-ups, 216 patients (62.2%) experienced recurrence, and 147 patients (42.4%) passed away. The 1-, 3-, and 5-year DFS rates were 69.8%, 41.5%, and 30.8%, and the 1-, 3-, and 5-year OS rates were 84.6%, 59.4%, and 52.2%, respectively. For the DFS rates, the P values of different groups of the NLR, PLR, GPR, ALR and FAR values were 0.088, 0.082, < 0.001, < 0.001 and < 0.001, respectively. For the OS rate, the P values of different groups of the NLR, PLR, GPR, ALR and FAR were 0.004, 0.008, < 0.001, < 0.001 and < 0.001, respectively (Fig. 3).

### Independent prognostic factors for DFS and OS rates

In the univariate analysis for DFS, variables with P < 0.1 including sex, age, portal vein invasion, ascites, AFP, tumor capsule, tumor size, tumor number, MVI, cell differentiation, MKI, NLR, PLR, GPR, ALR and FAR were selected for the multivariate analysis. According to the results of multivariate analysis, AFP > 400 ng/ml, ALR > 31, GPR > 0.48, MVI, absence of tumor capsule, and tumor size > 5 cm were independent prognostic factors for DFS (Table 2).

**Table 2:** Univariate and multivariate analysis of DFS

Variable		Univariate analysis		Multivariate analysis	
		HR (95%CI)	P value	HR (95%CI)	P value
Sex	Male/female	1.401 (0.947–2.072)	0.092		
Age (years)	≥ 60/ < 60	0.767 (0.560–1.050)	0.097		
HBsAg	Positive/negative	1.317 (0.874–1.985)	0.189		
Liver cirrhosis	Yes/no	1.080 (0.819–1.425)	0.586		
Portal vein invasion	Yes/no	2.710 (1.686–4.357)	< 0.001		
Ascites	Yes/no	2.997 (1.621–5.539)	< 0.001		
Ablation or TACE	Yes/no	0.833 (0.637–1.089)	0.181		
AFP (ng/ml)	> 400/ ≤ 400	1.674 (1.279–2.190)	< 0.001	1.377 (1.042–1.821)	0.025
Tumor capsule	No/yes	2.146 (1.497–3.078)	< 0.001	1.864 (1.289–2.697)	0.001
Tumor number	≥ 2/1	1.481 (1.006–2.181)	0.047		
Tumor size (cm)	> 5/≤ 5	1.766 (1.348–2.313)	< 0.001	1.440 (1.090–1.901)	0.010
MVI^a^	Yes/no	1.649 (1.259–2.160)	< 0.001	1.352 (1.022–1.789)	0.035
Cell differentiation	Moderate/well	2.248 (1.189–4.251)	0.013		
	Poor/well	2.869 (1.364–6.031)	0.005		
MKI^b^	No/yes	0.596 (0.395–0.900)	0.014		
Child class	B/A	1.683 (0.891–3.177)	0.109		
NLR	> 2.33/ ≤ 2.33	1.264 (0.965–1.656)	0.089		
PLR	> 117.09/ ≤ 117.09	1.270 (0.969–1.664)	0.083		
GPR	> 0.48/ ≤ 0.48	2.327 (1.770–3.060)	< 0.001	1.931 (1.445–2.581)	< 0.001
ALR	> 31/ ≤ 31	1.903 (1.455–2.488)	< 0.001	1.438 (1.083–1.910)	0.012
FAR	> 0.06/ ≤ 0.06	1.617 (1.234–2.117)	< 0.001		

^a^MVI: microvascular invasion

^b^MKI: multiple kinase inhibitor

In the univariate analysis for OS, variables with P < 0.1 including HBV, portal vein invasion, ascites, AFP, tumor capsule, tumor size, tumor number, MVI, cell differentiation, NLR, PLR, GPR, ALR and FAR, were then entered into the Cox multivariate analysis. The results demonstrated that PLR > 117.09, ALR > 31, GPR > 0.48, MVI, absence of tumor capsule, and tumor size > 5 cm were independent prognostic factors for OS (Table 3).

**Table 3:** Univariate and multivariate analysis of OS

Variable		Univariate analysis		Multivariate analysis	
		HR (95%CI)	P value	HR (95%CI)	P value
Sex	Male/female	1.478 (0.912–2.393)	0.113		
Age (years)	≥ 60/ < 60	0.670 (0.451–0.996)	0.048		
HBsAg	Positive/negative	1.630 (0.940–2.736)	0.083		
Liver cirrhosis	Yes/no	1.036 (0.742–1.448)	0.834		
Portal vein invasion	Yes/no	3.757 (2.256–6.256)	< 0.001		
Ascites	Yes/no	3.564 (1.804–7.039)	< 0.001		
Ablation or TACE	Yes/no	1.047 (0.757–1.449)	0.780		
AFP (ng/ml)	> 400/ ≤ 400	1.844 (1.334–2.549)	< 0.001		
Tumor capsule	No/yes	2.710 (1.811–4.056)	< 0.001	2.119 (1.394–3.222)	< 0.001
Tumor number	≥ 2/1	1.893 (1.230–2.915)	0.004		
Tumor size (cm)	> 5/ ≤ 5	2.170 (1.553–3.033)	< 0.001	1.646 (1.157–2.342)	0.006
MVI^a^	Yes/no	2.084 (1.495–2.906)	< 0.001	1.678 (1.195–2.355)	0.003
Cell differentiation	Moderate/well	2.718 (1.112–6.647)	0.028		
	Poor/well	3.703 (1.375–9.976)	0.010		
MKI^b^	No/yes	0.853 (0.492–1.480)	0.572		
Child class	B/A	1.380 (0.609–3.126)	0.440		
NLR	> 2.37/ ≤ 2.37	1.615 (1.157–2.253)	0.005		
PLR	> 117.09/ ≤ 117.09	1.542 (1.115–2.133)	0.009	1.465 (1.024–2.096)	0.037
GPR	> 0.48/ ≤ 0.48	3.002 (2.131–4.230)	< 0.001	2.554 (1.757–3.712)	< 0.001
ALR	> 31/ ≤ 31	2.255 (1.628–3.122)	< 0.001	1.553 (1.098–2.197)	0.013
FAR	> 0.06/ ≤ 0.06	1.984 (1.425–2.764)	< 0.001		

^a^MVI: microvascular invasion

^b^MKI: multiple kinase inhibitor

### The relationship between independent prognostic inflammatory markers and clinicopathological features

As the ALR and GPR were independent prognostic factors for DFS; and the ALR, GPR, and PLR were independent prognostic factors for OS, the correlations of common clinicopathological variables with different groups of the ALR, GPR and PLR were computed by the χ^2^ test. The results showed that the ALR was correlated with portal vein invasion, ascites, AFP, tumor number, tumor size, MVI, BCLC, AJCC and CNLC staging; the GPR was correlated with sex, portal vein invasion, ascites, AFP, tumor capsule, tumor number, tumor size, MVI, BCLC, AJCC and CNLC staging; and the PLR was correlated with sex, postoperative ablation or TACE, tumor capsule, tumor size, and BCLC staging (see Additional file 1).

### Creation and comparison of inflammatory scoring models for DFS and OS

We generated models for the ALR, GPR and PLR score. At the same time, we created an ALR-GPR score model for DFS, and the ALR-GPR, ALR-PLR, GPR-PLR and ALR-GPR-PLR score (A-G-P score) models for OS. For simplicity of calculation, ALR ≤ 31, GPR ≤ 0.48 and PLR ≤ 117.09 were defined as a score of 0, and ALR > 31, GPR > 0.48 and PLR > 117.09 were defined as a score of 1.

The ALR, GPR and PLR score models consisted of scores of 0 and 1, and the models for the ALR-GPR, ALR-PLR and GPR-PLR score consisted of scores of ≤ 1 and 2; the A-G-P score model consisted of scores of ≤ 1, 2 and 3 (Table 4).

**Table 4:** Models of inflammatory markers

Model for OS		Score	Model for DFS		Score
ALR score	ALR > 31	1	ALR score	ALR > 31	1
	ALR ≤ 31	0		ALR ≤ 31	0
GPR score	GPR > 0.48	1	GPR score	GPR > 0.48	1
	GPR ≤ 0.48	0		GPR ≤ 0.48	0
PLR score	PLR > 117.09	1	ALR-GPR score	ALR > 31 and GPR > 0.48	2
	PLR ≤ 117.09	0		others	≤ 1
ALR-GPR score	ALR > 31 and GPR > 0.48	2			
	others	≤ 1			
ALR-PLR score	ALR > 31 and PLR > 117.09	2			
	others	≤ 1			
GPR-PLR score	GPR > 0.48 and PLR > 117.09	2			
	others	≤ 1			
A-G-P score^a^	ALR > 31, GPR > 0.48 and PLR > 117.09	3			
	ALR > 31, GPR > 0.48, and PLR ≤ 117.09; or ALR > 31, PLR > 117.09, and GPR ≤ 0.48; or PLR > 117.09, GPR > 0.48, and ALR ≤ 31	2			
	Others	≤ 1			

^a^A-G-P score: ALR-GPR-PLR score

We further verified whether the above combined scoring models (as categorical variables) were independent predictors of prognosis through univariate and multivariate analyses. Obviously, the single inflammatory marker model ALR, GPR and PLR score were independent predictors. When verifying one combined scoring model, we no longer put the individual inflammatory markers that make up the model into analyses to exclude their interactions. The results demonstrated that all of the ALR-GPR, ALR-PLR, GPR-PLR and A-G-P score models were independent predictors for OS, and the ALR-GPR score was an independent predictive factor for DFS (see Additional files 2, 3, 4, 5, 6).

Then, we compared the single inflammatory marker models and the combined scoring models by the C-index, AIC and likelihood ratio. For OS, compared with other models, the A-G-P score model had the smallest AIC value (1569.94), the largest C-index value (0.653, 95% CI: 0.610–0.696) and the largest likelihood ratio (50.48), suggesting that the A-G-P score model has a better prediction accuracy, goodness of fit, and uniformity in predicting the survival of patients who underwent resection. In terms of DFS, the single inflammatory marker model, the GPR score had the smallest AIC value (2264.32), the largest C-index value (0.605, 95% CI: 0.572–0.638) and the largest likelihood ratio (37.39), suggesting that the GPR score model has a better prediction (Table 5).

**Table 5:** Comparison of models for OS/DFS

Model	AIC	C-index	Likelihood ratio
OS			
ALR score	1596.53	0.61	23.89
GPR score	1578.07	0.64	42.34
PLR score	1613.68	0.56	6.73
ALR-GPR score	1574.60	0.61	30.96
ALR-PLR score	1598.11	0.59	22.30
GPR-PLR score	1585.71	0.60	34.70
A-G-P score^a^	1569.94	0.65	50.48
DFS			
ALR score	2280.10	0.59	21.61
GPR score	2264.32	0.61	37.39
ALR-GPR score	2272.73	0.59	28.98

^a^A-G-P score: ALR-GPR-PLR score

### The effect of stratification of the A-G-P score model and GPR score model on different stages of HCC

**Figure. 4:**
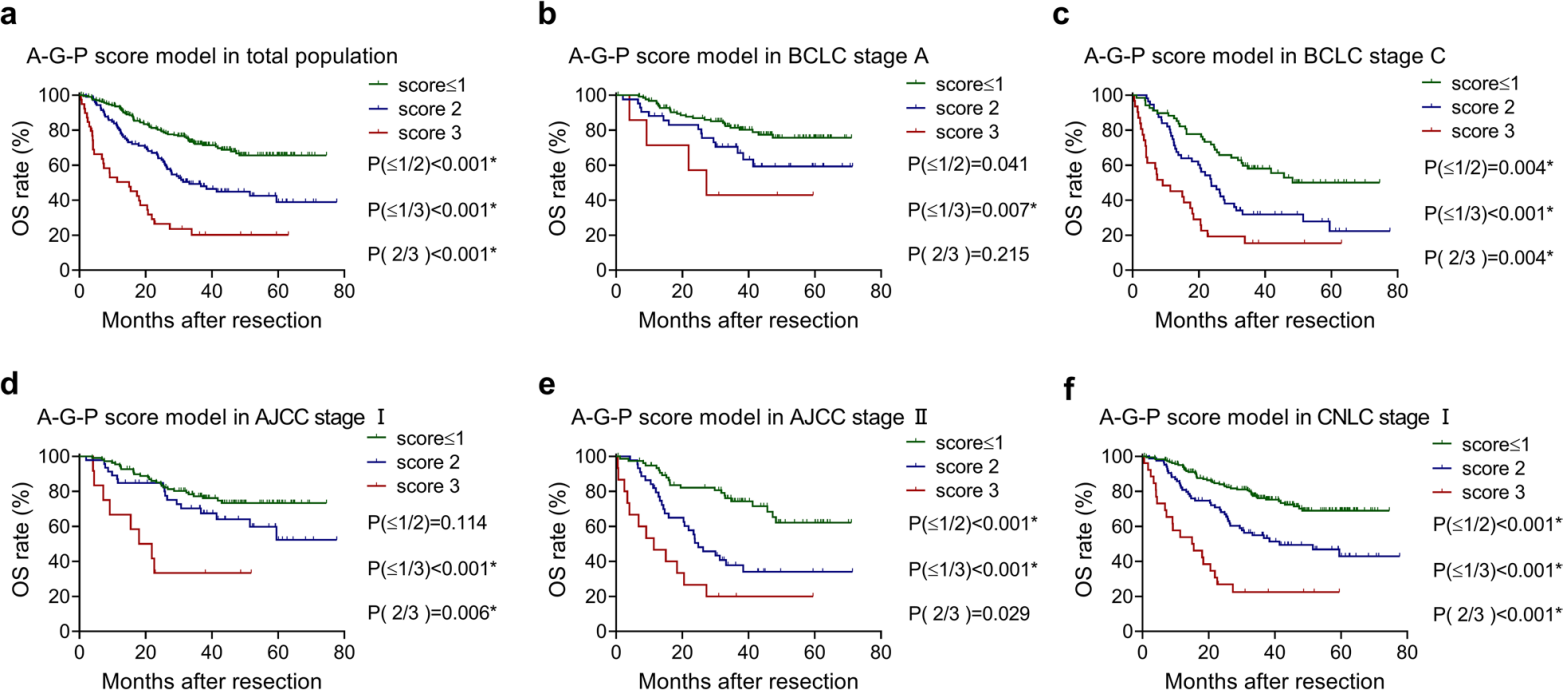
Kaplan–Meier analysis with log-rank test of A-G-P score model in (**a**) the total population(N_score≤_ _1_= 203, N_score2_ = 105, N_score3_ = 39), (**b**) BCLC stage A(N_score≤1_ = 126, N_score2_ = 42, N_score3_ = 7), (**c**) BCLC stage C(N_score≤1_ = 68, N_score2_ = 56, N_score3_ = 31), (**d**) AJCC stage I(N_score≤1_ = 108, N_score2_ = 46, N_score3_ = 12), (**e**) AJCC stage II(N_score≤1_ = 77, N_score2_ = 44, N_score3_ = 15) and (**f**) CNLC stage I(N_score≤1_ = 181, N_score2_ = 84, N_score3_ = 26) for OS. Asterisk indicates statistical difference

The A-G-P score model had a good discriminating ability for OS in the whole population with a statistically significant difference (score ≤ 1/score 2, P < 0.001; score ≤ 1/ score 3, P < 0.001; score 2/score 3, P < 0.001). For the pairwise comparison of the A-G-P score, we used Bonferroni correction, and P < 0.0167 (α = 0.05/3) was considered statistically significant between the different scores. Regarding BCLC staging, a score ≤ 1/score 3 (P = 0.007) in stage A; and a score ≤ 1/score 2 (P = 0.004), a score ≤ 1/score 3 (P < 0.001), and a score 2/score 3 (P < 0.004) in stage C were significantly different. Regarding AJCC staging, score ≤ 1/score 3 (P < 0.001) and score 2/score 3 (P = 0.006) in stage I, score ≤ 1/score 2 (P < 0.001) and score ≤ 1/score 3 (P < 0.001) in stage II were significantly different. For CNLC staging, a score ≤ 1/score 2 (P < 0.001), a score ≤ 1/score 3 (P < 0.001), and a score 2/score 3 (P < 0.001) in stage I showed significant differences for the prognosis of OS (Fig. 4).

**Figure. 5:**
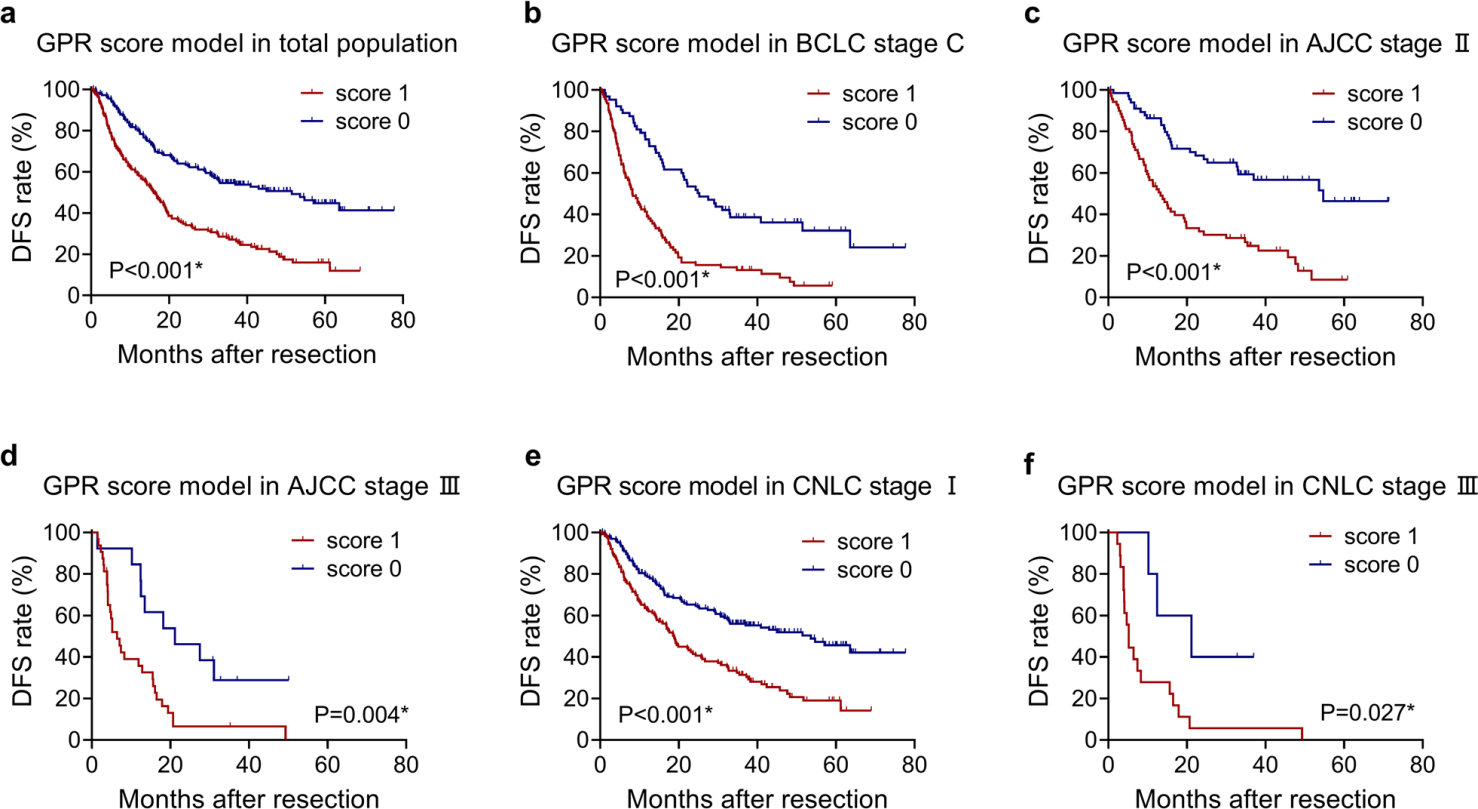
Kaplan–Meier analysis with log-rank test of GPR score model in (**a**) the total population(N_score1_ = 165, N_score0_ = 182), (**b**) BCLC stage C(N_score1_ = 92, N_score0_ = 63), (**c**) AJCC stage II(N_score1_ = 69, N_score0_ = 67), (**d**) AJCC stage III(N_score1_ = 32, N_score0_ = 13), (**e**) CNLC stage I(N_score1_ = 126, N_score0_ = 165) and (**f**) CNLC stage III(N_score1_ = 18, N_score0_ = 5) for DFS. Asterisk indicates statistical difference

The GPR score model was a good differentiator for DFS in the whole population, with P < 0.001. In BCLC stage C (P < 0.001), AJCC stage II (P < 0.001), AJCC stage III (P = 0.004), CNLC stage I (P < 0.001), and CNLC stage III (P = 0.027), different scores of the GPR score model showed significant differences for DFS (Fig. 5).

## Discussion

Cancer-related chronic inflammation is a remarkable characteristic of cancer and promotes the metastasis. The persistent presence of inflammatory cells in the tumor establishes cross-talk with tumor cells that may lead to the conversion of phenotypes to tumor support cells [23]. The long-term effect of HBV or HCV has a key impact on the development of HCC. At present, inflammatory scores have been suggested to have predictive value for the prognosis of HCC. The NLR and GPR have been verified as inflammation-related factors in predicting the survival of patients with HCC after liver resection [24].

The number of neutrophils has been independently correlated with TNM staging, performance status and poorer prognosis, indicating the key role of neutrophils in HCC [25]. High preoperative PLR values may be an adverse prognostic factor for OS and DFS in HCC patients. However, elevated PLR values are not highly associated with vascular invasion, tumor numbers, AFP levels or poor tumor grades [17].

In addition, patients with a high ALR have been shown to have an unsatisfactory prognosis, and the ALR is an independent prognostic factor for HCC [21]. In our study, an elevated ALR was associated with portal vein invasion, AFP levels, tumor numbers, tumor sizes and MVI, and was an independent predictor for both DFS and OS. It has been reported that an elevated FAR is associated with poor prognosis and higher relapse rates in HCC patients [22]; nevertheless, our study demonstrated that the FAR was not a prognostic factor in HCC.

Some studies have revealed that NLR is a predictive factor in HCC, but it was excluded after multivariate analysis in our research. To eliminate the possible influence of other inflammatory markers on NLR in the Cox multivariate analysis, we further conducted a Cox multivariate analysis with only NLR as an inflammatory marker and other significant univariate variables. This result supported our previous findings that the NLR was not an independent prognostic factor for OS (see Additional file 7). A retrospective study proposed that the inflammatory markers PLR and CRP (but not NLR) have prognostic value, possibly because they reflect the value of parameters representative of tumor growth and aggressiveness [26]. To some extent, these results are consistent with our research.

In the present study, inflammatory markers were found to be effective and reliable indicators for the prognosis of HCC [27], but the effect of inflammatory markers on prognosis in HCC after resection is still confusing. In our research, we examined the independent predictors of HCC patients with liver resection.

The ALR, GPR and PLR were independent prognostic factors for OS. Elevated serum AST values usually indicate extensive damage to the liver parenchyma and poor prognosis. The host's immune response to tumors depends on lymphocytes. Lymphopenia may lead to a poor lymphocyte-mediated tumor immune response, and a higher risk of cancer relapse [28]. Therefore, a higher ALR value predicts a worse prognosis. As a component of GPR, the elevation of γ-GT indicates poor liver function. Therefore, the higher γ-GT is, the greater the value of GPR, indicating poorer prognosis in HCC. In addition, another problem should be considered that the GPR and PLR are both independent factors affecting the survival of HCC. In calculating the GPR, the platelet count is in the denominator, which means that the larger the platelet count is, the smaller the value of GPR, indicating a better prognosis. However, in calculating the PLR, the platelet count is in the numerator, indicating that the larger the platelet count is, the worse the prognosis, which is contradictory to the results of GPR. Cancer cells can activate platelets, and the activation of platelets can lead to cancer-related inflammation, metastasis and cancer progression [29]. In contrast, platelets play important roles in cancer progression at different stages. For example, platelets suppress tumors via the downregulation of TC genes and induction of tumor cell apoptosis in early stages [30]. Thus, platelets have the dual roles of promoting tumor development and inhibiting tumor growth by influencing the tumor microenvironment and their powerful secretory function [31].

DFS has independent prognostic factors, including the ALR, the GPR and AFP. Notably, AFP is not considered an independent factor for OS, suggesting that a high AFP level (AFP > 400 ng/ml) has a greater effect on recurrence than on survival.

The A-G-P and GPR score models were selected as the optimal models for OS and DFS, respectively, by comparing the models using the AIC, C-index and likelihood ratio. These two models not only performed well in the total population but also showed good stratification ability for most of the different stages of HCC. In particular, in the A-G-P score model, 3 groups were significantly different in BCLC stage C and CNLC stage I, while 2 groups were statistically significant in AJCC stage I and AJCC stage II. We further analyzed why this scoring system performed well in both BCLC stage C (advanced stage) and CNLC stage I (early stage), and found that among the 155 patients with BCLC stage C, 132 patients were assigned to BCLC stage C simply because of their PS of 1 point. The PS point value is easily affected by the patient’s possible unclear expression and the subjectivity of the surgeon. CNLC stage I includes patients with PS values of 1 and 2 points, and 116 patients belonging to BCLC stage C will be reassigned to CNLC stage I according to the CNLC criterion. Thus, the above question will become easy to understand. With respect to the GPR score model for DFS, the model shows good discrimination ability in BCLC stage C, AJCC stage II, AJCC stage III, CNLC stage I and CNLC stage III.

Our research has room for improvements. First, the research was a single-center retrospective study, and there might be biases in sample selection. Next, portal hypertension probably has impacts on hematological indicators, but due to the lack of direct data on portal hypertension measurements, we did not take portal hypertension into consideration.

## Conclusions

At present, the prognosis of HCC is based mainly on staging systems and prognostic indicators, however, it is not sufficient to judge the prognosis of HCC by these criteria alone, because the prognosis of HCC is not only determined by the tumor itself, but also influenced by the patients' liver function. Our study focused on critical indexes of liver function and representative inflammatory cells, and found that changes in these markers have a significant impact on the prognosis of HCC.

In summary, our study demonstrated for the first time that the ALR-GPR-PLR score model was an independent predictor for OS and performed well in stratifying patients with HCC. We also proposed the GPR score model and confirmed its effect on DFS. These models are readily available and affordable, and could supplement and improve the existing prognostic criteria of HCC, providing guidance for postoperative interventions in patients with HCC.

## Supplementary Information

**Additional file 1**: The relationship between independent prognostic inflammatory markers.

**Additional file 2**: Univariate and multivariate analysis of OS for ALR-PLR score.

**Additional file 3**: Univariate and multivariate analysis of OS for ALR-GPR score.

**Additional file 4**: Univariate and multivariate analysis of OS for GPR-PLR score.

**Additional file 5**: Univariate and multivariate analysis of OS for A-G-P score.

**Additional file 6**: Univariate and multivariate analysis of DFS for ALR-GPR score.

**Additional file 7**: Univariate and multivariate analysis of OS for NLR.

## Data Availability

All data generated or analysed during this study are included in this published article (and its Additional files).

## Abbreviations

ALR: Aspartate aminotransferase-to-lymphocyte ratio
A-G-P score model: ALR-GPR-PLR score model
AIC: Akaike information criterion
C-index: Concordance index
DFS: Disease-free survival
FAR: Fibrinogen-to-albumin ratio
GPR: Gamma-glutamyl transpeptidase-to-platelet ratio
HCC: Hepatocellular carcinoma
NLR: Neutrophil-to-lymphocyte ratio
OS: Overall survival
PLR: Platelet-to-lymphocyte ratio
ROC: Receiver operating characteristic

## References

[1] Sung H, Ferlay J, Siegel RL, Laversanne M, Soerjomataram I, Jemal A, et al. Global cancer statistics 2020: GLOBOCAN estimates of incidence and mortality worldwide for 36 cancers in 185 countries. CA Cancer J Clin. 2021. 10.3322/caac.21660 33538338

[2] Chen W, Zheng R, Baade P, Zhang S, Zeng H, Bray F (2016). Cancer statistics in China, 2015. CA Cancer J Clin.

[3] Villanueva A, Hoshida Y, Battiston C, Tovar V, Sia D, Alsinet C (2011). Combining clinical, pathology, and gene expression data to predict recurrence of hepatocellular carcinoma. Gastroenterology.

[4] Bruix J, Gores G, Mazzaferro V (2014). Hepatocellular carcinoma: clinical frontiers and perspectives. Gut.

[5] Bruix J, Reig M, Sherman M (2016). Evidence-based diagnosis, staging, and treatment of patients with hepatocellular carcinoma. Gastroenterology.

[6] EASL Clinical Practice Guidelines (2018). Management of hepatocellular carcinoma. J Hepatol.

[7] Chun Y, Pawlik T, Vauthey J (2018). 8th Edition of the AJCC Cancer Staging Manual: pancreas and hepatobiliary cancers. Ann Surg Oncol.

[8] Xie D, Ren Z, Zhou J, Fan J, Gao Q (2020). 2019 Chinese clinical guidelines for the management of hepatocellular carcinoma: updates and insights. Hepatobiliary Surg Nutr.

[9] Han L, Lv Y, Guo H, Ruan Z, Nan K (2014). Implications of biomarkers in human hepatocellular carcinoma pathogenesis and therapy. World J Gastroenterol.

[10] Greten F, Grivennikov S (2019). Inflammation and Cancer: triggers, mechanisms, and consequences. Immunity.

[11] Zhang Y, Lu J, Du Y, Feng C, Wang L, Chen M (2018). Prognostic value of neutrophil-to-lymphocyte ratio and platelet-to-lymphocyte ratio in gastric cancer. Medicine.

[12] Hu Z, Tan S, Chen S, Qin S, Chen H, Qin S (2020). Diagnostic value of hematological parameters platelet to lymphocyte ratio and hemoglobin to platelet ratio in patients with colon cancer. Clin Chim Acta Int J Clin Chem..

[13] Ma J, Ke L, Liu Q (2018). The pretreatment platelet-to-lymphocyte ratio predicts clinical outcomes in patients with cervical cancer: a meta-analysis. Medicine.

[14] Zhao Z, Zhao X, Lu J, Xue J, Liu P, Mao H (2018). Prognostic roles of neutrophil to lymphocyte ratio and platelet to lymphocyte ratio in ovarian cancer: a meta-analysis of retrospective studies. Arch Gynecol Obstet.

[15] He C, Zhang Y, Cai Z, Lin X (2019). The prognostic and predictive value of the combination of the neutrophil-to-lymphocyte ratio and the platelet-to-lymphocyte ratio in patients with hepatocellular carcinoma who receive transarterial chemoembolization therapy. Cancer Manage Res.

[16] Uchinaka E, Amisaki M, Morimoto M, Tokuyasu N, Sakamoto T, Honjo S (2018). Utility and limitation of preoperative neutrophil lymphocyte ratio as a prognostic factor in hepatocellular carcinoma. Yonago Acta Med.

[17] Ma W, Zhang P, Qi J, Gu L, Zang M, Yao H (2016). Prognostic value of platelet to lymphocyte ratio in hepatocellular carcinoma: a meta-analysis. Sci Rep.

[18] Dai T, Deng M, Ye L, Liu R, Lin G, Chen X (2020). Prognostic value of combined preoperative gamma-glutamyl transpeptidase to platelet ratio and fibrinogen in patients with HBV-related hepatocellular carcinoma after hepatectomy. Am J Transl Res.

[19] Ke M, Zhang M, Su Q, Wei S, Zhang J, Wang Y (2018). Gamma-glutamyl transpeptidase to platelet ratio predicts short-term outcomes in hepatocellular carcinoma patients undergoing minor liver resection. J Surg Res.

[20] Zhao L, Yang D, Ma X, Liu M, Wu D, Zhang X (2019). The Prognostic Value of aspartate aminotransferase to lymphocyte ratio and systemic immune-inflammation index for overall survival of hepatocellular carcinoma patients treated with palliative treatments. J Cancer.

[21] Chen Y, He C, Wen T, Yan L, Yang J. The prognostic value of aspartate aminotransferase-to-lymphocyte ratio index in early-stage hepatocellular carcinoma after hepatectomy: a propensity-score matched analysis. Asia-Pacific J Clin Oncol. 2020. 10.1111/ajco.13458 33124200

[22] Xu Q, Yan Y, Gu S, Mao K, Zhang J, Huang P (2018). A novel inflammation-based prognostic score: the fibrinogen/albumin ratio predicts prognoses of patients after curative resection for hepatocellular carcinoma. J Immunol Res.

[23] Suarez-Carmona M, Lesage J, Cataldo D, Gilles C (2017). EMT and inflammation: inseparable actors of cancer progression. Mol Oncol.

[24] Wang Y, Sun K, Shen J, Li B, Kuang M, Cao Q (2019). Novel Prognostic Nomograms Based on Inflammation-Related Markers for Patients with Hepatocellular Carcinoma Underwent Hepatectomy. Cancer Res Treat Off J Korean Cancer Assoc.

[25] Margetts J, Ogle L, Chan S, Chan A, Chan K, Jamieson D (2018). Neutrophils: driving progression and poor prognosis in hepatocellular carcinoma?. Br J Cancer.

[26] Suner A, Carr B, Akkiz H, Uskudar O, Kuran S, Tokat Y, et al. Inflammatory markers C-reactive protein and PLR in relation to HCC characteristics. J Transl Sci. 2019;5(3). 10.15761/JTS.1000260 PMC6333412 30662766

[27] Huang L, Mo Z, Hu Z, Zhang L, Qin S, Qin X (2020). Diagnostic value of fibrinogen to prealbumin ratio and gamma-glutamyl transpeptidase to platelet ratio in the progression of AFP-negative hepatocellular carcinoma. Cancer Cell Int.

[28] Liao W, Zhang J, Zhu Q, Qin L, Yao W, Lei B (2014). Preoperative neutrophil-to-lymphocyte ratio as a new prognostic marker in hepatocellular carcinoma after curative resection. Transl Oncol.

[29] Palacios-Acedo A, Mège D, Crescence L, Dignat-George F, Dubois C, Panicot-Dubois L (2019). Platelets, thrombo-inflammation, and cancer: collaborating with the enemy. Front Immunol.

[30] Michael J, Wurtzel J, Mao G, Rao A, Kolpakov M, Sabri A (2017). Platelet microparticles infiltrating solid tumors transfer miRNAs that suppress tumor growth. Blood.

[31] Plantureux L, Mege D, Crescence L, Carminita E, Robert S, Cointe S (2020). The interaction of platelets with colorectal cancer cells inhibits tumor growth but promotes metastasis. Cancer Res.

