# Supplementary material for: Prognostic value of preoperative inflammatory markers in patients with hepatocellular carcinoma who underwent curative resection
Source: Cancer Cell Int. 2021 Sep 17;21:500. doi: 10.1186/s12935-021-02204-3 (PMC8447627; doi:10.1186/s12935-021-02204-3)
Supplement: Supplementary file 1 — Additional file 1: The relationship between independent prognostic inflammatory markers. [file 12935_2021_2204_MOESM1_ESM.docx]

| **Additional file 1.** The relationship between independent prognostic inflammatory markers  and clinicopathological features | | | | | | | | | | |
| --- | --- | --- | --- | --- | --- | --- | --- | --- | --- | --- |
|  |  |  |  |  |  |  |  |  |  |  |
| Variable | | ALR | | P value | GPR | | P value | PLR | | P value |
|  |  | >31 | ≤31 |  | >0.48 | ≤0.48 |  | >117.09 | ≤117.09 |  |
| Sex | Male | 112 | 178 | 0.085 | 148 | 142 | 0.003* | 106 | 184 | 0.043* |
|  | Female | 29 | 28 |  | 17 | 40 |  | 29 | 28 |  |
| Age(years) | ≥60 | 35 | 60 | 0.377 | 44 | 51 | 0.777 | 30 | 65 | 0.086 |
|  | <60 | 106 | 146 |  | 121 | 131 |  | 105 | 147 |  |
| HBsAg | Positive | 126 | 174 | 0.191 | 146 | 154 | 0.293 | 116 | 184 | 0.818 |
|  | Negative | 15 | 32 |  | 19 | 28 |  | 19 | 28 |  |
| Liver cirrhosis | Yes | 96 | 126 | 0.187 | 110 | 112 | 0.320 | 80 | 142 | 0.144 |
|  | No | 45 | 80 |  | 55 | 70 |  | 55 | 70 |  |
| Portal vein invasion | Yes | 15 | 6 | 0.003* | 17 | 4 | 0.002* | 10 | 11 | 0.398 |
|  | No | 126 | 200 |  | 148 | 178 |  | 125 | 201 |  |
| Ascites | Yes | 9 | 2 | 0.012* | 9 | 2 | 0.021* | 7 | 4 | 0.163 |
|  | No | 132 | 204 |  | 156 | 180 |  | 128 | 208 |  |
| Ablation or TACE | Yes | 80 | 108 | 0.429 | 88 | 100 | 0.763 | 83 | 105 | 0.029* |
|  | No | 61 | 98 |  | 77 | 82 |  | 52 | 107 |  |
| AFP(ng/ml) | >400 | 68 | 64 | 0.001* | 74 | 58 | 0.013* | 56 | 76 | 0.292 |
|  | ≤400 | 73 | 142 |  | 91 | 124 |  | 79 | 136 |  |
| Tumor capsule | Yes | 121 | 183 | 0.402 | 138 | 166 | 0.033* | 112 | 192 | 0.036* |
|  | No | 20 | 23 |  | 27 | 16 |  | 23 | 20 |  |
| Tumor number | ≥2 | 23 | 19 | 0.047* | 27 | 15 | 0.021* | 18 | 24 | 0.575 |
|  | 1 | 118 | 187 |  | 138 | 167 |  | 117 | 188 |  |
| Tumor size(cm) | >5 | 82 | 86 | 0.003* | 93 | 75 | 0.005* | 84 | 84 | <0.001* |
|  | ≤5 | 59 | 120 |  | 72 | 107 |  | 51 | 128 |  |
| MVI^a^ | Yes | 80 | 92 | 0.027* | 97 | 75 | 0.001* | 71 | 101 | 0.368 |
|  | No | 61 | 114 |  | 68 | 107 |  | 64 | 111 |  |
| MKI^b^ | Yes | 14 | 19 | 0.826 | 16 | 17 | 0.910 | 12 | 21 | 0.753 |
|  | No | 127 | 187 |  | 149 | 165 |  | 123 | 191 |  |
| Child class | A | 133 | 202 | 0.117 | 156 | 179 | 0.053 | 130 | 205 | 1.000 |
|  | B | 8 | 4 |  | 9 | 3 |  | 5 | 7 |  |
| Cell differentiation | Poor | 19 | 16 | 0.193 | 18 | 17 | 0.170 | 18 | 17 | 0.260 |
|  | Moderate | 112 | 171 |  | 138 | 145 |  | 107 | 176 |  |
|  | Well | 10 | 19 |  | 9 | 20 |  | 10 | 19 |  |
| BCLC staging | A | 47 | 128 | <0.001* | 62 | 113 | <0.001* | 55 | 120 | 0.01* |
|  | B | 7 | 10 |  | 11 | 6 |  | 6 | 11 |  |
|  | C | 87 | 68 |  | 92 | 63 |  | 74 | 81 |  |
| AJCC staging | Ⅰ | 58 | 108 | 0.01* | 64 | 102 | <0.001* | 59 | 107 | 0.257 |
|  | Ⅱ | 56 | 80 |  | 69 | 67 |  | 54 | 82 |  |
|  | Ⅲ | 27 | 18 |  | 32 | 13 |  | 22 | 23 |  |
| CNLC staging | Ⅰ | 108 | 183 | 0.004* | 126 | 165 | 0.001* | 109 | 182 | 0.344 |
|  | Ⅱ | 17 | 16 |  | 21 | 12 |  | 14 | 19 |  |
|  | Ⅲ | 16 | 7 |  | 18 | 5 |  | 12 | 11 |  |

a: MVI: microvascular invasion b: MKI: multiple kinase inhibitor

*statistical difference
