# Supplementary material for: Prognostic value of preoperative inflammatory markers in patients with hepatocellular carcinoma who underwent curative resection
Source: Cancer Cell Int. 2021 Sep 17;21:500. doi: 10.1186/s12935-021-02204-3 (PMC8447627; doi:10.1186/s12935-021-02204-3)
Supplement: Supplementary file 3 — Additional file 3: Univariate and multivariate analysis of OS for ALR-GPR score. [file 12935_2021_2204_MOESM3_ESM.docx]

**Additional file 3**. Univariate and multivariate analysis of OS for ALR-PLR score

| Variable |  | Univariate analysis | | Multivariate analysis | |
| --- | --- | --- | --- | --- | --- |
|  |  | HR(95%CI) | P value | HR(95%CI) | P value |
| Sex | Male/ Female | 1.478 (0.912-2.393) | 0.113 |  |  |
| Age(years) | ≥60/<60 | 0.670 (0.451-0.996) | 0.048 |  |  |
| HBsAg | Positive/ Negative | 1.630 (0.940-2.736) | 0.083 |  |  |
| Liver cirrhosis | Yes/ No | 1.036 (0.742-1.448) | 0.834 |  |  |
| Portal vein invasion | Yes/ No | 3.757 (2.256-6.256) | <0.001 |  |  |
| Ascites | Yes/ No | 3.564 (1.804-7.039) | <0.001 |  |  |
| Ablation or TACE | Yes/ No | 1.047 (0.757-1.449) | 0.780 |  |  |
| AFP(ng/ml) | >400/≤400 | 1.844 (1.334-2.549) | <0.001 |  |  |
| Tumor capsule | No/Yes | 2.710 (1.811-4.056) | <0.001 | 2.132 (1.407-3.231) | <0.001 |
| Tumor number | ≥2/1 | 1.893 (1.230-2.915) | 0.004 |  |  |
| Tumor size(cm) | >5/≤5 | 2.170 (1.553-3.033) | <0.001 | 1.585 (1.118-2.249) | 0.010 |
| MVI^a^ | Yes/ No | 2.084 (1.495-2.906) | <0.001 | 1.651 (1.174-2.320) | 0.004 |
| Cell differentiation | Moderate/ well | 2.718 (1.112-6.647) | 0.028 |  |  |
|  | Poor/well | 3.703 (1.375-9.976) | 0.010 |  |  |
| MKI^b^ | No/Yes | 0.853 (0.492-1.480) | 0.572 |  |  |
| Child class | B/A | 1.380 (0.609-3.126) | 0.440 |  |  |
| NLR | >2.37/≤2.37 | 1.615 (1.157-2.253) | 0.005 |  |  |
| GPR | >0.48/≤0.48 | 3.002 (2.131-4.230) | <0.001 | 2.697 (1.903-3.823) | <0.001 |
| FAR | >0.06/≤0.06 | 1.984 (1.425-2.764) | <0.001 |  |  |
| ALR-PLR score | score2/score≤1 | 2.425 (1.716-3.428) | <0.001 | 2.128 (1.489-3.042) | <0.001 |

a: MVI: microvascular invasion b: MKI: multiple kinase inhibitor
