# Supplementary material for: Prognostic value of preoperative inflammatory markers in patients with hepatocellular carcinoma who underwent curative resection
Source: Cancer Cell Int. 2021 Sep 17;21:500. doi: 10.1186/s12935-021-02204-3 (PMC8447627; doi:10.1186/s12935-021-02204-3)
Supplement: Supplementary file 6 — Additional file 6: Univariate and multivariate analysis of DFS for ALR-GPR score. [file 12935_2021_2204_MOESM6_ESM.docx]

**Additional file 6**. Univariate and multivariate analysis of DFS for ALR-GPR score

| Variable |  | Univariate analysis | | Multivariate analysis | |
| --- | --- | --- | --- | --- | --- |
|  |  | HR(95%CI) | P value | HR(95%CI) | P value |
| Sex | Male/ Female | 1.401 (0.947-2.072) | 0.092 |  |  |
| Age(years) | ≥60/<60 | 0.767 (0.560-1.050) | 0.097 |  |  |
| HBsAg | Positive/ Negative | 1.317 (0.874-1.985) | 0.189 |  |  |
| Liver cirrhosis | Yes/ No | 1.080 (0.819-1.425) | 0.586 |  |  |
| Portal vein invasion | Yes/ No | 2.710 (1.686-4.357) | <0.001 |  |  |
| Ascites | Yes/ No | 2.997 (1.621-5.539) | <0.001 |  |  |
| Ablation or TACE | Yes/ No | 0.833 (0.637-1.089) | 0.181 |  |  |
| AFP(ng/ml) | >400/≤400 | 1.674 (1.279-2.190) | <0.001 |  |  |
| Tumor capsule | No/Yes | 2.146 (1.497-3.078) | <0.001 | 1.802 (1.248-2.604) | 0.002 |
| Tumor number | ≥2/1 | 1.481 (1.006-2.181) | 0.047 |  |  |
| Tumor size(cm) | >5/≤5 | 1.766 (1.348-2.313) | <0.001 | 1.585 (1.205-2.086) | 0.001 |
| MVI^a^ | Yes/ No | 1.649 (1.259-2.160) | <0.001 | 1.498 (1.138-1.972) | 0.004 |
| Cell differentiation | Moderate/ Poor | 0.784 (0.508-1.210) | 0.272 |  |  |
|  | Well/ Poor | 0.349 (0.166-0.733) | 0.005 |  |  |
| MKI^b^ | No/Yes | 0.596 (0.395-0.900) | 0.014 | 0.629 (0.416-0.952) | 0.028 |
| Child class | B/A | 1.683 (0.891-3.177) | 0.109 |  |  |
| NLR | >2.37/≤2.37 | 1.264 (0.965-1.656) | 0.089 |  |  |
| PLR | >117.09/≤117.09 | 1.270 (0.969-1.664) | 0.083 |  |  |
| FAR | >0.06/≤0.06 | 1.617 (1.234-2.117) | <0.001 |  |  |
| ALR-GPR score | score2/score≤1 | 2.257 (1.702-2.994) | <0.001 | 2.089 (1.569-2.781) | <0.001 |

a: MVI: microvascular invasion b: MKI: multiple kinase inhibitor
